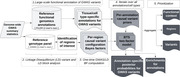# Multi‐omic cell type‐level inference of mechanisms underlying non‐coding genetic variants for Alzheimer’s disease

**DOI:** 10.1002/alz.086500

**Published:** 2025-01-03

**Authors:** Pavel P. Kuksa, Luke Carter, Jeffrey Cifello, Yuk Yee Leung, Li‐San Wang

**Affiliations:** ^1^ Penn Neurodegeneration Genomics Center, Dept of Pathology and Laboratory Medicine, University of Pennsylvania, Philadelphia, PA USA; ^2^ Penn Neurodegeneration Genomics Center, Perelman School of Medicine, University of Pennsylvania, Philadelphia, PA USA

## Abstract

**Background:**

Recent Alzheimer’s disease (AD) genome‐wide association studies () have identified >75 risk loci, with >98% of genome‐wide significant variants residing in non‐coding genomic regions, making it more difficult to infer their functional contexts. In this study, we aim to *jointly* 1) fine‐map causal loci/variants, and 2) identify affected cell types and functional elements by interrogating large‐scale collections of thousands of heterogenous, cell type‐specific functional genomic (FG) datasets.

**Method:**

We analyzed the full genome‐wide summary statistics (n = 21,101,114 variants) from the recent AD GWAS (Bellenguez et al, 2022) (Ncases = 111,326, Ncontrols = 677,663). The variants were analyzed using the recently introduced Bayesian framework (Bayesian tissue score, BTS https://bitbucket.org/wanglab‐upenn/workspace/projects/BTS) on >70,000 harmonized genome‐wide cell‐type level FG tracks (FILER; Kuksa et al, 2022), including those for FANTOM5 enhancers, Roadmap ChromHMM enhancers, ENCODE DNase hypersensitivity sites and active histone marks, and EpiMap enhancers (4,225 tracks). Using BTS, we calculated Bayesian prior odds for each of the annotation tracks overlapped with genome‐wide GWAS signals and then computed annotation‐specific causal variant and loci posteriors. With this systematic, hypothesis‐free multi‐omic approach, we jointly analyzed and ranked AD‐relevant loci, variants, and their functional contexts (affected cell types and corresponding genomic features).

**Result:**

The BTS pipeline identified 131 non‐HLA genomic regions with 49,049 variants, across 1,294 overlapping annotation tracks on 640 cell types, with up to two distinct causal signals in each locus. BTS prioritized 152 annotations tracks with >2 prior odds (3.60 causal prior odds average) across 108 cell types belonging to the blood (57), brain (21), heart (12), digestive (12), (6) tissue categories. In total, 94 genomic loci and 119 variants prioritized in these cell types (posterior > .5).

**Conclusion:**

Large‐scale evaluation of AD GWAS data provides insights into functional contexts and potentially causal biological mechanisms of AD.